# Human-Centered Baby Massage Mobile App for Low Birth Weight Infants: Usability Testing and a Pre-Post Pilot Intervention Study in Indonesia

**DOI:** 10.2196/73153

**Published:** 2026-09-30

**Authors:** Supriatin Supriatin, Regidor III Dioso, Hafizah Che Hassan

**Affiliations:** 1 STIKes Cirebon Cirebon, Jawa Barat Indonesia; 2 Lincoln University College Petailing Jaya Malaysia

**Keywords:** low birth weight, baby massage, mobile health, human-centered design, infant growth

## Abstract

**Background:**

Low birth weight (LBW) is associated with increased risks of neonatal morbidity, developmental delays, and long-term health problems. Although baby massages have been shown to improve growth and neurodevelopmental outcomes in LBW infants, access to suitable training and adherence to massage protocols remain challenging, particularly in low-resource settings. Mobile health interventions may help address these gaps by providing scalable and standardized education on infant massage.

**Objective:**

This study aimed to design and evaluate a human-centered baby massage mobile app to support the growth and development of LBW infants in Indonesia.

**Methods:**

A human-centered iterative design approach was used to develop the mobile app. Usability was assessed using the System Usability Scale among 42 caregivers of LBW infants. Qualitative data were collected through semistructured interviews and analyzed thematically to understand user experience. A pilot pre-post intervention study was conducted to evaluate the impact of the app on caregivers’ knowledge, confidence, adherence to baby massage practices, and infant growth outcomes. Pre- and postintervention measures were compared.

**Results:**

The app demonstrated high usability, with a mean System Usability Scale score of 78.6 (SD 8.2), indicating good usability (≥68). Most participants rated the app as “excellent” or “good.” Qualitative findings highlighted improved caregiver confidence and ease of learning massage techniques. Statistically significant improvements were observed in caregiver knowledge (mean difference 20.5 points; *P*=.002), confidence (21.6 points; *P*=.001), and adherence to recommended massage practices (20.3 points; *P*=.02). Infant outcomes also improved, including weight gain (mean increase 330 g; *P*=.003) and head circumference (mean increase 1.3 cm; *P*=.004).

**Conclusions:**

The human-centered baby massage mobile app showed promising feasibility and effectiveness in improving caregiver knowledge, confidence, and adherence to infant massage practices. The app provides a culturally appropriate and user-friendly tool to support early interventions for LBW infants in resource-limited settings.

## Introduction

Low birth weight (LBW), defined by the World Health Organization as a birthweight less than 2500 g, remains a substantial global public health challenge [1]. LBW is a critical indicator of neonatal morbidity and mortality, with an estimated 15% to 20% of all births worldwide classified as LBW, representing more than 20 million births annually [2]. The burden of LBW is prevalent in South Asia and sub-Saharan Africa, with 44% and 28% of LBW cases globally, respectively [3]. In Indonesia, the prevalence of LBW ranges from approximately 6% to 10% of all live births, according to national health surveys and global estimates [4,5]. LBW can pose several health risks for infants, such as stunted growth, neurodevelopmental delays, and an increased risk of infections and chronic diseases [6,7]. Such challenges underscore the urgent need for effective interventions to mitigate the long-term sequelae associated with LBW.

Existing interventions for LBW infants can be divided into methods such as kangaroo mother care (KMC) [8,9] and those that use modern technology [10-12]. KMC refers to holding the infant in skin-to-skin contact with the caregiver and has been widely adopted in low- and middle-income countries because of its practicality and effectiveness [8]. However, its reliance on continuous physical contact can be impractical for caregivers, especially in resource-limited settings. Complementary nonpharmacological techniques, including baby massages, have potential benefits, but this procedure usually necessitates training and routine practice, which often remain inaccessible [13].

Baby massages, a form of touch intervention, have shown promise in promoting health outcomes for LBW infants. Research indicates that massage can enhance weight gain, support neurodevelopment, and strengthen the emotional bond between infants and caregivers [14-18]. Randomized controlled trials and systematic reviews further suggest that infant massage significantly improves weight gain and neurobehavioral development in LBW infants [19-22]. Despite these benefits, barriers such as a lack of trained health care providers and inconsistent app by caregivers limit its widespread adoption.

The therapeutic benefits of baby massages in neonatal care are well documented. As a nonpharmacological approach, it promotes vagal nerve stimulation and facilitates improved digestion and increased weight gain [23]. Moreover, it promotes neurodevelopment through enhanced sensory integration and modulation of stress hormones [18,20,24]. Baby massages promote emotional bonding, which is crucial for the psychological development of the infant [22]. Studies have demonstrated that it enhances digestive and immune functions and reduces levels of stress hormones in infants and parents alike [23,24]. It is also involved in critical motor and sensory development, especially in premature and term infants [16]. However, in low- and middle-income countries, structural challenges make it difficult to find and use baby massages, including health care infrastructure and sociocultural obstacles [25]. The diversity of massage techniques and cultural practices also creates challenges in its implementation, highlighting the importance of standardized training and evidence-based guidelines [26,27]. In Indonesia, the limited availability of relevant and culturally sensitive educational materials has been a major obstacle to its adoption.

The rise of mobile health (mHealth) has shown promise in improving parental knowledge and compliance with neonatal care practices [28,29]. Parents have been encouraged to practice KMC and monitor infant growth through some mobile apps [11,12]. Such tools have also been shown to support prenatal care, breastfeeding, and early childhood development, and to improve parental confidence, adherence to health recommendations, and child health outcomes [29-31]. Studies show that mobile apps with interactive and user-centered features can be associated with higher user satisfaction and improved maternal health behaviors [32]. However, despite the increasing use of mHealth technologies, there is a significant lack of literature describing mHealth for baby massage education, particularly in multiculturally diverse contexts [33].

In many cases, existing mHealth apps are unable to implement human-centered design principles vital for usability and engagement, particularly in the Indonesian context [34]. Additionally, few evidence-based, culturally appropriate interventions exist to address the specific needs of LBW infants and their caregivers [35,36]. Little is currently known about the factors influencing the digital classroom experience in this field, including opportunities for bias against visual assets, as most research around baby massage education and experience continues to rely on face-to-face instructional culture. This creates a major gap in knowledge concerning how effective mHealth apps can be in increasing the knowledge, skills, and confidence of parents to perform baby massages [33]. Evidence is limited regarding infant health outcomes or how usability features may influence user engagement, satisfaction, and long-term adherence [34].

Filling these gaps is essential for informing scalable, accessible, and effective interventions targeting diverse populations. To ensure the development of such clinically meaningful mobile apps, we need a user-centered design process, complemented with a solid evaluation framework [34]. This study aims to address these gaps by developing and evaluating a human-centered baby massage mobile app designed to enhance growth and development in LBW infants in Indonesia.

## Methods

### Study Design

This study used a mixed methods design consisting of (1) usability testing and (2) a single-arm pilot pre-post intervention study conducted over 4 weeks.

The usability phase evaluated user experience (UX) and system performance using the System Usability Scale (SUS) and qualitative interviews.

The pilot phase used a 1-group pretest-posttest design to assess changes in caregiver knowledge, confidence, adherence to baby massage practices, and infant growth parameters following exposure to the mobile app.

No control group was included in this pilot study, as the primary objective was to assess the feasibility, usability, and preliminary effectiveness.

### Participant Recruitment and Sample Size Rationale

Participants were recruited between January and March 2025 through community health centers, pediatric outpatient clinics, and online announcements distributed via maternal health networks in West Java, Indonesia.

Eligibility criteria included the following: being a primary caregiver (mother or father) of an infant with documented LBW (<2500 g), infant age between 0 and 12 months, ownership of an Android smartphone, and ability to read Bahasa Indonesia.

Caregivers of infants with congenital abnormalities or severe medical instability were excluded.

The sample size (N=42) was determined based on recommendations for pilot feasibility studies, which suggest 30 to 50 participants to estimate preliminary effect sizes and usability metrics. The sample was also considered adequate for detecting moderate pre-post effects using paired statistical testing.

### Overview

A human-centered iterative design framework guided the development and evaluation of a mobile app designed to optimize growth and development in LBW infants through baby massages. The app design and development process was broken down into 6 clear steps, each contributing to the app definition and validation process.

### Stages of App Design and Development

#### Overview

The development of the baby massage mobile app followed a systematic, human-centered design framework, resulting in a functional and user-friendly tool. The process was iterative, consisting of six development cycles that produced six progressively refined versions of the app. Each iteration incorporated user feedback to improve usability, content, and functionality. A brief description of each stage is presented in the subsections below.

#### Stage 1: Establishing the Study Team

A multidisciplinary team was established, comprising neonatologists, pediatricians, child development experts, mobile app developers, and UX designers. The team also included pediatric nurse specialists, nursing researchers, and software engineers.

Regular meetings were conducted to ensure alignment among team members, foster interdisciplinary collaboration, and integrate clinical insights with technical perspectives. These meetings also facilitated knowledge exchange across disciplines and supported systems thinking, which helped define the project’s scope, objectives, and technical approach.

#### Stage 2: Defining a Set of App Design Requirements

The app design requirements were informed by a comprehensive literature review and expert consultations. Academic databases were systematically searched for peer-reviewed articles, systematic reviews, and clinical guidelines related to LBW infants, baby massages, and mHealth apps (PubMed and Google Scholar; publications up to October 2023). Records published since 2019 were emphasized to ensure relevance and alignment with current best practices. We extracted findings on effective interventions, user preferences, and technological features and identified gaps in the literature, specifically for culturally tailored digital solutions for LBW infants in low-resource settings. These efforts were further complemented by consultations with neonatologists, pediatricians, child development specialists, and UX designers to inform screen design requirements. Several key features emerged: step-by-step tutorials for baby massages, progress tracking, reminders, and culturally relevant content. These requirements were based on evidence-based practices and adapted to the specific needs of LBW infants and their caregivers [37].

#### Stage 3: Defining User Requirements

Focus group discussions (FGDs) were conducted with caregivers of LBW infants and health care providers to identify user requirements. The discussions explored users’ preferences, challenges, and expectations regarding a baby massage app.

Participants emphasized the need for a simple and intuitive interface, clear visual guidance, and offline functionality, particularly to accommodate limited internet access in rural settings [38].

#### Stage 4: Creating the App Prototype

In addition to instructional videos, interactive guides were incorporated to enhance user engagement. These guides allowed caregivers to access additional information, such as diagrams and animations, to better understand massage techniques and their benefits. The design emphasized intuitive navigation and responsive feedback to support ease of use.

A growth tracker feature was also included to enable caregivers to monitor infants’ development. The dashboard displayed key indicators such as weight and head circumference in an easy-to-read format, allowing users to visualize progress over time.

The prototype was developed as a set of wireframes representing the app’s layout and structure. Design tools such as Figma (Figma, Inc), Adobe XD (Adobe Inc), and Sketch (Sketch BV) were used to visualize the interface, typography, and icons through semi-interactive mock-ups.

#### Stage 5: Expert UX Review

A panel of experts in neonatal care, digital health, and UX design conducted a comprehensive review of the prototype. The panel included neonatologists with expertise in LBW infant care, clinicians experienced in mHealth apps in low-resource settings, and UX designers specializing in usability and user engagement.

The review process was structured and began with an initial briefing session, during which the prototype’s features, design principles, and intended user journey were presented. Experts then evaluated the prototype based on predefined criteria, including usability, functionality, visual design, sustainability, and cultural relevance.

Feedback from the experts led to refinements in several areas, including navigation clarity, instructional content, feature integration (eg, instructional videos and dashboard), and cultural appropriateness for the target Indonesian users.

#### Stage 6: Testing the Prototype

Usability testing and pilot testing were conducted to assess the refined prototype in real-world conditions.

### Usability Testing

We conducted usability testing with 42 caregivers of LBW infants recruited from a tertiary care hospital in Indonesia. Respondents who were willing to download and evaluate the app were included in the study sample. To evaluate the app’s usability, we used the SUS, which produces scores from 0 to 100. A higher score indicates improved usability. Furthermore, qualitative feedback on UX was collected via semistructured interviews [39]. We provided participants the app prototype and asked them to perform tasks such as finding a tutorial, setting reminders, and tracking their progress. Observations of users’ interactions with the app, along with feedback collected through the SUS and interviews, were analyzed. Quantitative data from the SUS were analyzed using descriptive statistics, and thematic analysis was applied to the qualitative data.

### Pilot Testing Phase

The pilot study used a single-arm pre-post design and was conducted among 42 caregivers of LBW infants from various socioeconomic backgrounds in Indonesia.

Knowledge, confidence, and adherence to baby massage practices were assessed using preintervention and postintervention questionnaires. The instrument, known as the Caregiver Baby Massage Knowledge, Confidence, and Adherence Questionnaire, was developed by the research team, comprising neonatologists, pediatricians, child development specialists, and nursing researchers.

The questionnaire development followed an iterative process, including a literature review on baby massage practices and caregiver self-efficacy, as well as expert consultation in neonatal care and digital health. The final instrument consisted of 30 items across three domains:

1. Knowledge (10 items)—assessing caregivers’ understanding of baby massage techniques, benefits, and precautions
2. Confidence (10 items)—measuring caregivers’ perceived ability to correctly and consistently perform baby massages
3. Adherence (10 items)—evaluating compliance with recommended baby massage routines

Items in the confidence and adherence domains were scored on a 5-point Likert scale ranging from 1 (strongly disagree) to 5 (strongly agree). Knowledge items were scored as 1 (incorrect) or 5 (correct). Domain scores were calculated by summing item scores, with higher scores indicating better knowledge, confidence, or adherence.

The questionnaire was translated into Bahasa Indonesia and validated in a preliminary study involving 30 caregivers. Reliability testing demonstrated good internal consistency, with Cronbach α values of 0.88 (knowledge), 0.90 (confidence), and 0.86 (adherence).

### Intervention Procedure

During the 4-week intervention period, caregivers were instructed to use the mobile app as their primary guidance tool for baby massage practice. Specifically, caregivers were asked to perform the following tasks:

- Watch instructional baby massage videos within the app at least 3 times per week
- Perform baby massage sessions at home at least once daily (minimum 5 sessions per week)
- Log massage completion in the app after each session
- Record infant weight weekly using digital baby scales available at community health centers
- Enter infant growth measurements, including weight and head circumference, into the in-app growth tracking feature

The app provided automated daily reminders to encourage adherence. All instructional materials were accessible offline after initial download.

No biochemical or laboratory biomarkers were assessed. Infant outcomes were limited to anthropometric measurements, specifically weight and head circumference.

Information regarding caregiver responses, app use patterns, and infant growth parameters was collected at baseline and after completion of the 4-week intervention. The mobile app for baby massages was developed as a functional prototype (Figure 1).

**Figure 1:**
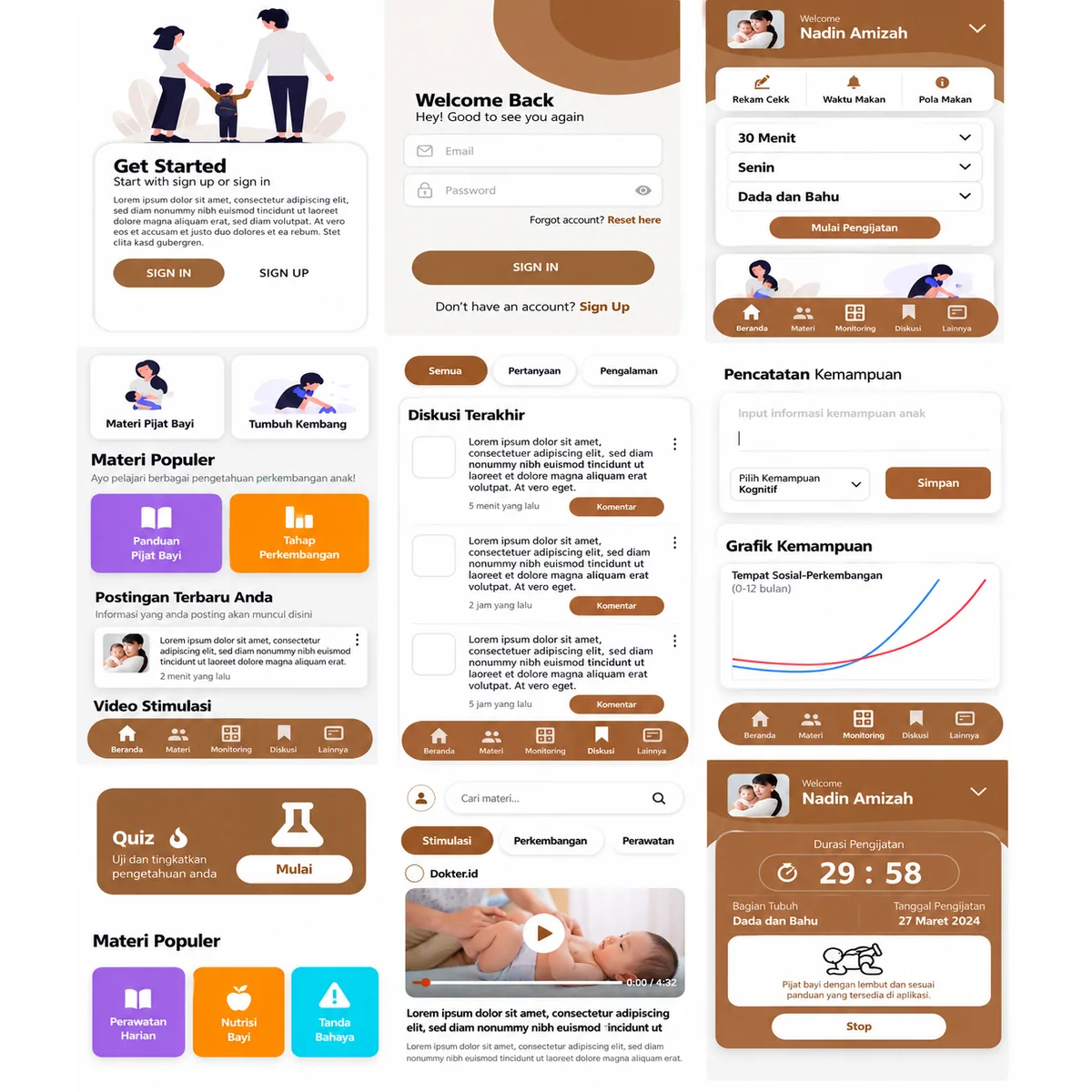
Mobile app for baby massages.

### Statistical Analysis

Paired *t* tests (2-tailed) were conducted to compare preintervention and postintervention outcomes for caregiver knowledge, confidence, adherence scores, and infant growth parameters. Statistical significance was set at *P*<.05.

### Ethical Considerations

This study was approved by the institutional review board of STIKep PPNI Jawa Barat (III/045/KEPK/STIKep/PPNI/Jabar/XI/2024). All participants provided written informed consent prior to participation. Participants did not receive financial compensation for their participation in the study. Caregivers were informed of their right to withdraw at any time without consequences. All collected data were anonymized and stored securely in password-protected databases.

## Results

### Demographic Characteristics of Participants

The study included 42 caregivers of infants aged 0 to 12 months. The demographic characteristics of the participants are presented in Table 1. Most of the participants (n=35, 83.3%) were mothers with an average age of 29.5 (SD 5.2) years. Most participants had a university-level education (n=31, 73.8%) and were first-time caregivers (n=25, 59.5%). Nearly all participants (n=40, 95.2%) had no prior experience with baby massage.

**Table 1:** Demographic characteristics of participants (N=42).

Characteristic			Values, n (%)
Gender			
	Female	35 (83.3)	
	Male	7 (16.7)	
Age (years)			
	<25	10 (23.8)	
	25-34	27 (64.3)	
	≥35	5 (11.9)	
Education level			
	High School	8 (19)	
	University	31 (73.8)	
	Postgraduate	3 (7.1)	
First-time caregiver			25 (59.5)
Prior baby massage experience			
	Yes	2 (4.8)	
	No	40 (95.2)	

### Usability Testing Results

Table 2 presents the descriptive statistics for the SUS scores. The mean SUS score was 78.6 (SD 8.2), indicating a high level of usability. Most participants (85%) rated the app as “excellent” or “good” for ease of use and navigation. The highest-rated aspect was “ease of learning” (mean score 4.5; 5-point scale), while the lowest-rated aspect was “visual design appeal” (mean score 3.9; 5-point scale; Table 2).

**Table 2:** Descriptive statistics for usability testing using the System Usability Scale.

Usability metric	Value
Overall System Usability Scale score, mean (SD)	78.6 (8.2)
Ease of navigation, mean (SD)	4.3 (0.7)
Ease of learning, mean (SD)	4.5 (0.6)
Visual design appeal, mean (SD)	3.9 (0.8)
Task completion rate (%)	92
Error rate (%)	5.2

### Qualitative Feedback From Users

Thematic analysis identified key themes related to usability, functionality, aesthetic design, user satisfaction, and cultural relevance. Table 3 summarizes the themes, categories, and example quotes.

**Table 3:** Thematic analysis of user feedback.

Theme and category			Example quote
Usability			
	Ease of use	“I found the app very easy to navigate, and I didn’t need much time to learn how to use it.”	
	Clarity of features	“The app’s instructions are clear, but some icons could be more intuitive.”	
	Intuitive navigation	“The app is very easy to use. I found the tutorials and reminders without any difficulty.”	
Functionality			
	Reminder system	“The reminder feature is very helpful in ensuring I don’t forget my baby’s checkups.”	
	Tracking progress	“I like how I can track my baby’s weight and feeding schedule in one place.”	
	Informative content	“The videos and interactive guides taught me how to massage my baby properly. I feel more confident now.”	
Aesthetic design			
	Visual appeal	“The design is good, but a more colorful interface would make it more engaging.”	
User satisfaction			
	Overall experience	“I would recommend this app to other parents; it makes managing my baby’s health much easier.”	
Cultural relevance			
	Local language support	“I appreciate that the app is in Bahasa Indonesia. It makes it easier for me to understand.”	
	Culturally appropriate	“The massage techniques shown in the app are similar to what my mother taught me. It feels familiar and comfortable.”	

### Pilot Testing

Caregivers demonstrated a significant increase in knowledge about baby massage techniques and their benefits, with a mean improvement of 20.5 points (*P*=.002). Confidence levels among caregivers improved significantly, with a mean increase of 21.6 points (*P*=.001). Caregivers reported feeling more capable of performing baby massage correctly and consistently. Adherence to baby massage practices improved by 20.3 points (*P*=.02), indicating that caregivers were more likely to follow the recommended massage routines after using the app. Infants showed significant improvements in weight (mean increase of 330 g; *P*=.003) and head circumference (mean increase of 1.3 cm; *P*=.004), suggesting positive effects of the intervention on infant growth and development (Table 4).

**Table 4:** Preintervention and postintervention outcomes for caregivers and infants.

Outcome measure	Preintervention, mean (SD)	Postintervention, mean (SD)	Mean difference (SD)	*P* value
Caregiver knowledge	65.2 (8.5)	85.7 (6.3)	20.5 (7.1)	.002
Caregiver confidence	60.8 (9.2)	82.4 (7.8)	21.6 (8.3)	.001
Adherence to practices	55.3 (10.1)	75.6 (8.9)	20.3 (9.5)	.02
Infant weight (grams)	2200 (150)	2530 (140)	330 (120)	.003
Infant head circumference (cm)	32.5 (1.2)	33.8 (1.1)	1.3 (0.9)	.004

## Discussion

### Principal Findings

This study assessed the effectiveness of a human-centered mobile app designed to support the growth and development of LBW infants in Indonesia through baby massages. The app achieved a high usability rating, reflecting its user-friendly design and ease of navigation.

Postintervention evaluations showed significant improvements in caregivers’ knowledge (*P*=.002), confidence (*P*=.001), and adherence to baby massage practices (*P*=.02). Importantly, infants whose caregivers used the app demonstrated significant increases in weight (*P*=.003) and head circumference (*P*=.004), indicating positive effects on their growth.

The app’s high usability and positive user feedback highlight its success in addressing the needs of its target audience. Caregivers praised its intuitive interface, clear instructions, and culturally relevant content, which were designed to reduce cognitive load and enhance engagement. Features such as the reminder system and progress tracking were particularly valued, as they supported consistent massage routines and enabled caregivers to monitor infant growth. These findings align with human-centered design principles, which emphasize tailoring digital health solutions to users’ needs and contexts [33,34,40,41].

While the app’s usability was generally high, minor concerns regarding visual design and icon intuitiveness suggest areas for future improvement. This study builds on these insights by introducing a culturally tailored, human-centered app specifically designed for Indonesian caregivers, addressing a gap in the literature.

The findings corroborate prior research on the benefits of baby massages and mHealth interventions for LBW infants. For instance, Vickers et al [16] reported that baby massages enhance weight gain and neurodevelopment in LBW infants, while Knop et al [28] highlighted the role of mHealth interventions in improving parental education and adherence to neonatal care practices. Similarly, Bangor et al [42] found that high usability scores in health apps are associated with increased user satisfaction and adherence to health recommendations.

Additionally, the app incorporates traditional massage techniques that align with local practices, making it more acceptable and practical for Indonesian caregivers, thus enhancing its relevance and effectiveness [36]. Furthermore, the app’s offline functionality addresses the challenge of limited internet access in rural areas, where nearly 80% of the population lacks reliable connectivity. This approach aligns with global health strategies advocating for digital tools to improve maternal and child health outcomes, especially in resource-constrained settings.

This study, while demonstrating promising outcomes, is not without limitations. One notable constraint is the relatively small sample size, which may restrict the generalizability of the findings. Additionally, the research was conducted within a specific cultural and regional context in Indonesia, potentially limiting its applicability to other settings with different cultural norms and health care environments. To address these limitations, future studies should aim to include larger and more diverse participant groups, as well as evaluate the app’s usability and effectiveness across varied cultural contexts. Another limitation is the short duration of the intervention, which prevents conclusions about the long-term impact of the app on infant growth and development. Extending the study period and incorporating follow-up assessments would provide deeper insights into the sustained benefits of the app. Furthermore, while qualitative feedback offered valuable perspectives on UXs, a more structured and comprehensive qualitative analysis could uncover additional insights into caregiver preferences and areas for improvement.

This pilot study used a single-arm pre-post design without a control group; therefore, causal inference cannot be established. Improvements in infant growth may have been influenced by natural growth progression or other unmeasured factors. Future randomized controlled trials are needed to confirm effectiveness.

### Conclusions

This study highlights the effectiveness of a culturally tailored, human-centered baby massage mobile app in improving caregiver knowledge, confidence, and adherence to infant massage practices, while also positively influencing the growth and development of LBW infants in Indonesia. The app’s high usability reflects its user-friendly design and intuitive navigation. The integration of such digital health tools into routine postnatal care offers a promising strategy for addressing the challenges faced by caregivers of LBW infants, particularly in low-resource settings. The app’s culturally relevant content, local language support, and offline functionality make it accessible and practical for users in diverse contexts, including rural areas with limited internet access. Positive feedback from participants further emphasizes the app’s value, with caregivers appreciating features such as step-by-step tutorials, progress tracking, and reminders. While minor usability concerns, such as the need for a more visually engaging interface, were noted, overall satisfaction with the app was high, indicating its potential as a reliable tool for supporting infant care. Looking ahead, future research should focus on scaling up the app, conducting longer-term evaluations to assess its sustained impact, and exploring its integration into existing neonatal care programs.

## Abbreviations

KMC: kangaroo mother care
LBW: low birth weight
mHealth: mobile health
SUS: System Usability Scale
UX: user experience
